# Automatic acromegaly detection using deep learning on hand images: a multicenter observational study

**DOI:** 10.1210/clinem/dgag027

**Published:** 2026-02-27

**Authors:** Yuka Ohmachi, Mizuho Nishio, Ichiro Abe, Kunihisa Kobayashi, Tomoko Iida, Manabu Shirakawa, Yuichi Nagata, Kazuhito Takeuchi, Akira Taguchi, Yasuyuki Kinoshita, Noriaki Fukuhara, Hiroshi Nishioka, Shigeyuki Tahara, Shingo Fujio, Takafumi Ogura, Masamichi Kurosaki, Yurika Hada, Shinji Susa, Yuki Otsuka, Fumio Otsuka, Ikuhiro Ishida, Hiraku Kameda, Kenichi Oyama, Shozo Yamada, Masaki Kobatake, Yuka Oi-Yo, Genki Fujii, Seiji Tomofuji, Yuriko Sasaki, Hironori Bando, Masaaki Yamamoto, Genzo Iguchi, Yuma Motomura, Yasutaka Tsujimoto, Naoki Yamamoto, Masaki Suzuki, Shin Urai, Michiko Takahashi, Takamichi Murakami, Wataru Ogawa, Hidenori Fukuoka

**Affiliations:** Division of Diabetes and Endocrinology, Department of Internal Medicine, Kobe University Graduate School of Medicine, Kobe 650-0017, Hyogo, Japan; Department of Radiology, Kobe University Graduate School of Medicine, Kobe 650-0017, Hyogo, Japan; Department of Endocrinology and Diabetes Mellitus, Fukuoka University Chikushi Hospital, Chikushino 818-8502, Fukuoka, Japan; Department of Endocrinology and Diabetes Mellitus, Fukuoka University Chikushi Hospital, Chikushino 818-8502, Fukuoka, Japan; Department of Neurosurgery, Hyogo Medical University, Nishinomiya 663-8501, Hyogo, Japan; Department of Neurosurgery, Hyogo Medical University, Nishinomiya 663-8501, Hyogo, Japan; Department of Neurosurgery, Nagoya University Graduate School of Medicine, Nagoya 466-8560, Aichi, Japan; Department of Neurosurgery, Nagoya University Graduate School of Medicine, Nagoya 466-8560, Aichi, Japan; Department of Neurosurgery, Graduate School of Biomedical and Health Sciences, Hiroshima University, Hiroshima 734-8551, Hiroshima, Japan; Department of Neurosurgery, Graduate School of Biomedical and Health Sciences, Hiroshima University, Hiroshima 734-8551, Hiroshima, Japan; Department of Hypothalamic and Pituitary Surgery, Toranomon Hospital, Minato-ku 105-8470, Tokyo, Japan; Department of Hypothalamic and Pituitary Surgery, Toranomon Hospital, Minato-ku 105-8470, Tokyo, Japan; Department of Neurological Surgery, Nippon Medical School Musashikosugi Hospital, Kawasaki 211-8533, Kanagawa, Japan; Department of Neurosurgery, Graduate School of Medical and Dental Sciences, Kagoshima University, Kagoshima 890-8520, Kagoshima, Japan; Division of Neurosurgery, Department of Brain and Neurosciences, Faculty of Medicine, Tottori University, Yonago 683-8504, Tottori, Japan; Division of Neurosurgery, Department of Brain and Neurosciences, Faculty of Medicine, Tottori University, Yonago 683-8504, Tottori, Japan; Department of Neurology, Hematology, Metabolism, Endocrinology, and Diabetology, Faculty of Medicine, Yamagata University, Yamagata 990-9585, Yamagata, Japan; Department of Neurology, Hematology, Metabolism, Endocrinology, and Diabetology, Faculty of Medicine, Yamagata University, Yamagata 990-9585, Yamagata, Japan; Department of General Medicine, Okayama University Graduate School of Medicine, Dentistry, and Pharmaceutical Sciences, Okayama 700-8558, Okayama, Japan; Department of General Medicine, Okayama University Graduate School of Medicine, Dentistry, and Pharmaceutical Sciences, Okayama 700-8558, Okayama, Japan; Division of Diabetes and Endocrinology, Hyogo Prefectural Kakogawa Medical Center, Kakogawa 675-8555, Hyogo, Japan; Department of Rheumatology, Endocrinology, and Nephrology, Faculty of Medicine and Graduate School of Medicine, Hokkaido University, Sapporo 060-8638, Hokkaido, Japan; Department of Neurosurgery, International University of Health and Welfare Mita Hospital, Minato-ku 108-8329, Tokyo, Japan; Department of Hypothalamic and Pituitary Surgery, Toranomon Hospital, Minato-ku 105-8470, Tokyo, Japan; Neurosurgery Center, Moriyama Memorial Hospital, Edogawa-ku 134-0081, Tokyo, Japan; Division of Diabetes and Endocrinology, Department of Internal Medicine, Kobe University Graduate School of Medicine, Kobe 650-0017, Hyogo, Japan; Division of Diabetes and Endocrinology, Department of Internal Medicine, Kobe University Graduate School of Medicine, Kobe 650-0017, Hyogo, Japan; Division of Diabetes and Endocrinology, Department of Internal Medicine, Kobe University Graduate School of Medicine, Kobe 650-0017, Hyogo, Japan; Division of Diabetes and Endocrinology, Department of Internal Medicine, Kobe University Graduate School of Medicine, Kobe 650-0017, Hyogo, Japan; Division of Diabetes and Endocrinology, Department of Internal Medicine, Kobe University Graduate School of Medicine, Kobe 650-0017, Hyogo, Japan; Division of Diabetes and Endocrinology, Department of Internal Medicine, Kobe University Graduate School of Medicine, Kobe 650-0017, Hyogo, Japan; Division of Diabetes and Endocrinology, Department of Internal Medicine, Kobe University Hospital, Kobe 650-0017, Hyogo, Japan; Division of Diabetes and Endocrinology, Department of Internal Medicine, Kobe University Graduate School of Medicine, Kobe 650-0017, Hyogo, Japan; Division of Diabetes and Endocrinology, Department of Internal Medicine, Kobe University Graduate School of Medicine, Kobe 650-0017, Hyogo, Japan; Faculty of Clinical Nutrition and Dietetics, Department of Clinical Nutrition and Dietetics, Konan Women's University, Kobe 658-0001, Hyogo, Japan; Division of Diabetes and Endocrinology, Department of Internal Medicine, Kobe University Graduate School of Medicine, Kobe 650-0017, Hyogo, Japan; Division of Diabetes and Endocrinology, Department of Internal Medicine, Kobe University Graduate School of Medicine, Kobe 650-0017, Hyogo, Japan; Division of Diabetes and Endocrinology, Department of Internal Medicine, Kobe University Graduate School of Medicine, Kobe 650-0017, Hyogo, Japan; Division of Diabetes and Endocrinology, Department of Internal Medicine, Kobe University Graduate School of Medicine, Kobe 650-0017, Hyogo, Japan; Division of Diabetes and Endocrinology, Department of Internal Medicine, Kobe University Graduate School of Medicine, Kobe 650-0017, Hyogo, Japan; Division of Diabetes and Endocrinology, Department of Internal Medicine, Kobe University Hospital, Kobe 650-0017, Hyogo, Japan; Department of Nutrition, Kobe University Hospital, Kobe 650-0017, Hyogo, Japan; Department of Radiology, Kobe University Graduate School of Medicine, Kobe 650-0017, Hyogo, Japan; Division of Diabetes and Endocrinology, Department of Internal Medicine, Kobe University Graduate School of Medicine, Kobe 650-0017, Hyogo, Japan; Division of Diabetes and Endocrinology, Department of Internal Medicine, Kobe University Hospital, Kobe 650-0017, Hyogo, Japan

**Keywords:** acromegaly, early detection, deep learning, artificial intelligence, hand images

## Abstract

**Context:**

Acromegaly poses clinical challenges in terms of early diagnosis and intervention. Therefore, the development of novel diagnostic tools is essential. Although artificial intelligence (AI) models based on external appearance have been proposed, privacy concerns have limited their use.

**Objective:**

To develop a privacy-conscious deep learning model for detecting acromegaly using hand images.

**Methods:**

This nationwide multicenter study enrolled 716 patients (317 with acromegaly and 399 controls) and 11 480 images from 15 Japanese pituitary centers. The inclusion criteria were age ≥18 years and care received at the participating facilities. Hand images focusing on the dorsal and fist sign, excluding the palm/fingerprint regions, were used to develop the model. The data were split into training/validation (12 centers) and test (3 centers) datasets. A ResNet-50-based model was trained using PyTorch with data augmentation and 5-fold cross-validation. For each patient, the predictions were averaged over 4 images. The performance of the model was compared with that of endocrinologists.

**Results:**

The model achieved a sensitivity of 0.89, specificity of 0.91, positive predictive value of 0.88, negative predictive value of 0.93, F1-score of 0.89, and an area under the receiver operating characteristic curve of 0.96, outperforming specialists (F1-score range: 0.43-0.63).

**Conclusion:**

This study highlights the utility of dorsal hand and fist sign as diagnostic clues for acromegaly, which the AI model captured more accurately than endocrinologists. Using this privacy-conscious feature, this model can be deployed in public settings like health checkups. Further validation using larger datasets, including healthy individuals and diverse diseases, is necessary.

Acromegaly is a disorder characterized by excessive secretion of growth hormone (GH), resulting in characteristic facial features and limb enlargement (1). Without appropriate treatment, mortality is 2 to 3 times higher than that in the general population, and life expectancy is reduced by approximately a decade (2, 3). Advances in surgical techniques, pharmacotherapy, and radiotherapy have significantly improved patient survival in recent years (4); however, improvements in post-treatment quality of life (QoL) remain limited. Further progress is essential to address this ongoing clinical burden, particularly in terms of early diagnosis and timely therapeutic intervention. Although acromegaly is a rare disease with a prevalence of 8 to 24 per 100 000 individuals, it is encountered in general practice because of its various symptoms (5). Acral enlargement, headache, and facial changes are common symptoms (6). However, its gradual progression often prevents patients from recognizing body changes, making early diagnosis particularly challenging (7, 8). Patients typically first consult non-endocrinology departments, including orthopedics, dental and maxillofacial surgery, gynecology, gastroenterology, and cardiology. Initial suspicion was raised by non-endocrine specialists in 37.2% of patients and by general practitioners in 16.2% of patients (9). Consequently, referrals to endocrinologists are frequently delayed, and approximately 24% of patients experience diagnostic delays exceeding 10 years. These delays are associated with increased complications, higher mortality, and diminished QoL (10).

With the emergence of AI, particularly neural networks capable of approximating any function from data, novel opportunities have emerged in medical diagnostics (11). Neural networks have demonstrated proficiency in classifying images, natural language, and audio signals, thereby facilitating advancements in various medical applications, such as differentiating between types of skin cancer (12) and identifying abnormalities in endoscopic images (13). Although previous studies have explored the use of facial image data for diagnosing acromegaly (14-17), this approach has not yet been adopted clinically, perhaps partly because of concerns regarding personal identification. In this study, we shifted the focus from the face to the hand as an anatomical area that poses challenges for personal identification in AI-based screening. Hand and foot enlargement is observed in 90% of patients with acromegaly and is considered a particularly common physical characteristic (6, 18). Because the palmar surface contains fingerprints, we restricted the inputs to the dorsum of the hand and the clenched fist regions regarded as less identifying and therefore more socially acceptable for image-based screening. Consistent with data protection principles, facial images and fingerprints warrant heightened safeguards. Although palmprint recognition (19) and near-infrared vein biometrics have been described, dorsal-hand or clenched-fist images obtained under conventional visible light are not typically regarded as definitive biometric identifiers. Although dorsal knuckle crease patterns vary among individuals and have been explored for recognition (20), their use remains uncommon in practice. Our aim was to develop an AI model capable of detecting slight physical manifestations of acromegaly in a privacy-conscious manner, with potential applicability in non-specialist settings, where earlier recognition can improve clinical outcomes. This concept was further supported by previous clinical reports that identified the “fist sign” as a characteristic feature of acromegaly, defined as the inability to cover the fingernails with the center of the palm when clenching the fist (21). Although a recent study reported an AI model using hand images for acromegaly diagnosis (22), it did not specifically address privacy concerns, distinguishing our approach from ethical considerations for real-world implementation of the model. By leveraging this sign, we aimed to construct a diagnostic model capable of identifying visual abnormalities in hand images. Although the diagnosis of acromegaly often depends on subjective visual impressions, standardized criteria for these assessments are lacking. In this study, we aimed to develop an AI-based model that holds promise for real-world deployment. As part of this effort, we investigated whether the fist sign, which has not previously been validated as a useful physical finding for diagnosing acromegaly, could be established as a potential clinical feature of the disease, as it proved valuable as an input for our AI-driven detection.

## Materials and methods

### Study design and patient selection

This multicenter observational nationwide study was approved by the Institutional Review Board of Kobe University Hospital (#B230135) and was performed in accordance with the Declaration of Helsinki. Written informed consent was obtained from 726 patients; however, one patient withdrew consent, resulting in 725 patients participating in the study. This study was conducted with the support of the Pituitary Patient Advocacy Group of Japan as part of a Patient and Public Involvement initiative.

### Datasets

Patients were recruited from 15 medical facilities between December 27, 2023 and December 31, 2024 (Supplemental Table 1) (23). Participants were prospectively enrolled as individuals with acromegaly or as controls.

Acromegaly was diagnosed based on Japanese guideline (24). Briefly, the criteria require the presence of at least one major clinical feature, such as enlargement of the hands and feet, typical facial changes, or macroglossia, along with a lack of GH suppression after a 75 g oral glucose load, elevated serum insulin-like growth factor I (IGF-I) levels adjusted for age and sex, and radiological evidence of a pituitary adenoma on Magnetic Resonance Imaging.

The inclusion criteria for the acromegaly cohort were as follows: (1) age ≥18 years at the time of consent, (2) receiving treatment at any of the participating facilities, (3) diagnosis of acromegaly confirmed by a specialist, and (4) provision of written informed consent by the patient. The inclusion criteria for the control group were that participants were determined by an endocrinologist not to have acromegaly and that they met criteria (1), (2), and (4) used for the acromegaly cohort. The control group included many patients who were assessed for pituitary disorders and were determined not to have acromegaly based on GH and IGF-I levels. Additionally, the group included individuals who were judged by experienced endocrinologists, based on a comprehensive clinical assessment, to be highly unlikely to have acromegaly, even in the absence of hormone testing. Although we did not systematically record the detailed medical histories of the control group, all participants were selected based on expert clinical evaluations. IGF-I measurements were not performed in all control participants because of resource constraints and ethical considerations. These individuals did not exhibit characteristic physical findings or complications suggestive of acromegaly and subjecting them to additional testing was deemed unnecessary and potentially burdensome. The exclusion criteria for both cohorts were as follows: (1) deemed by the principal investigator as unsuitable for inclusion in the study and (2) presence of wounds covering more than one-third of the hand.

**Figure 1:**
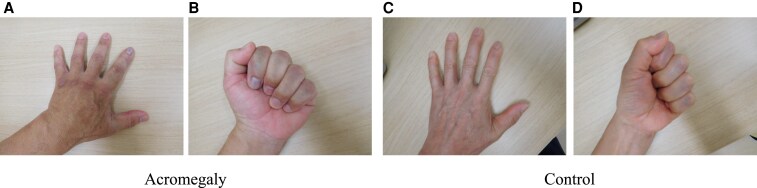
Representative hand images (dorsal hand and fist sign) from participants with acromegaly and control participants. Representative examples of hand images of patients with acromegaly (panels A and B; left two panels) and control participants (panels C and D; right two panels). Both open-hand (A and C) and clenched-fist (B and D) postures are shown for each group. Two types of hand images are captured: a frontal image of the hand in an extended position (dorsal side; A and C) and a frontal image of the hand in a clenched position with the thumb positioned externally (B and D). Images used to train and evaluate an artificial intelligence (AI) model to detect the characteristic features of acromegaly based on hand morphology.

Demographic data, specifically age and sex, were collected from all participants. Additionally, 2 types of hand images were captured: a frontal image of the hand in an extended position (dorsal side) and an image of the hand in a clenched position with the thumb positioned externally (Fig. 1). These were defined as the “dorsal hand sign” and the “fist sign,” respectively. Multiple images were captured when possible.

For patients with acromegaly, the following additional data were collected: height; weight; history of surgery for acromegaly; postoperative remission status (remission or residual); duration of remission; use of pharmacological therapy; GH and IGF-I levels (closest to the date of image acquisition and preoperative values); and regular replacement therapy with hydrocortisone, sex hormones, or levothyroxine. In recent clinical guidelines, biochemical remission of acromegaly is defined based on IGF-I levels normalized for age and sex (25). Therefore, the IGF-I Standard Deviation Score (SDS) < 2 was used as a criterion for remission in this analysis. The cohort was partitioned at the facility level, and approximately 80% of the patients were assigned to the training/validation dataset and 20% to the test dataset, maintaining site exclusivity between the splits.

### Network architecture

In this study, we executed a series of processes using Python (https://www.python.org/), including data preprocessing, model construction, training, and evaluation, to develop a deep learning model for acromegaly detection from hand images. The PyTorch framework (https://pytorch.org/) was used for model development and inference, with ResNet-50 pretrained on ImageNet as the backbone network.

For image preprocessing, a standardized procedure was applied to all the images used for training and evaluation. Initially, the orientation of the hand in each image was adjusted such that the wrist was positioned below the midpoint of the image. To protect participant anonymity, we assessed the necessity of additional anonymization measures (eg, background masking or noise injection) and determined that they were unnecessary because all images were prospectively captured to depict only the hands against a uniform background and contained no personally identifying elements. Therefore, to preserve image fidelity for downstream analyses, we did not apply background overpainting (filling) or artificial noise to the images. Subsequently, each image was resized to fit within a predefined size of 784 × 784 pixels while maintaining its original aspect ratio. The resized image was then placed at the center of a white-background canvas. For images with an alpha channel (RGBA), the transparency information was managed appropriately during the compositing process. Finally, all images were saved in the JPEG format. This preprocessing pipeline standardizes images with diverse backgrounds, sizes, and orientations into a uniform format that is suitable for model training.

**Figure 2:**
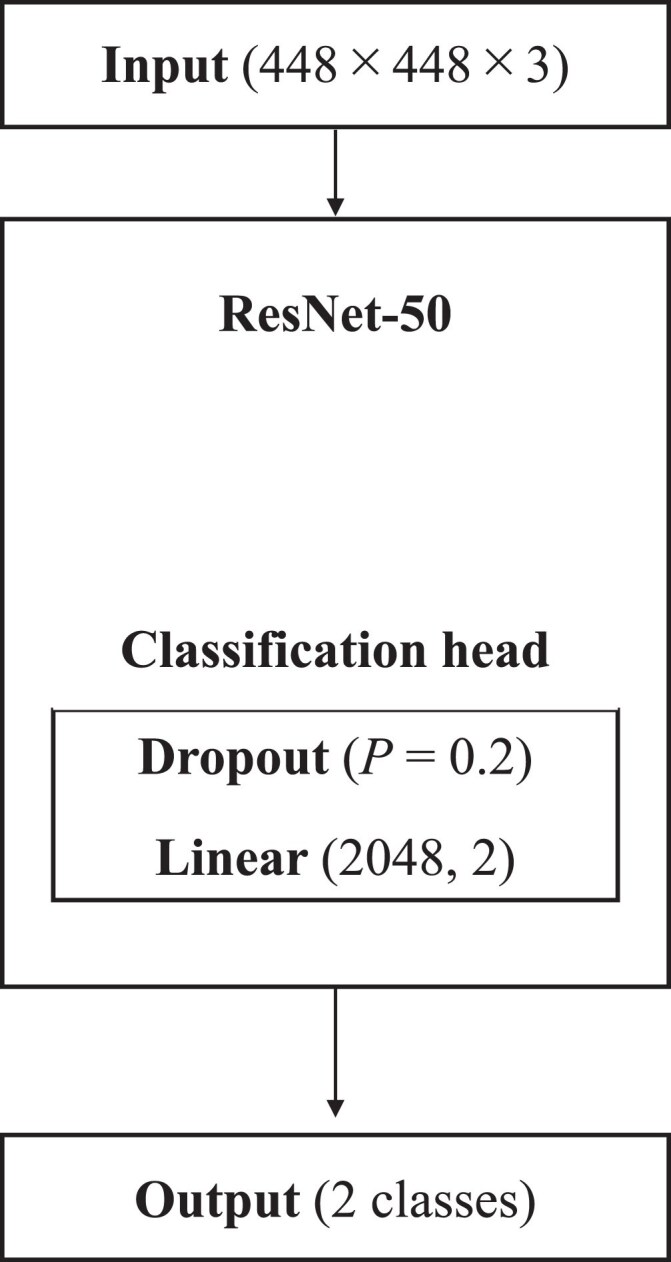
Architecture of the modified ResNet-50 model. Schematic of the modified ResNet-50 model used for binary classification. The original fully connected (fc) layer of ResNet-50 was removed and replaced with a custom classification head consisting of a dropout layer (dropout probability *P* = .2), followed by a linear layer (2048 input features to two output classes). The input images were resized to 448 × 448 pixels, and the model was optimized for a two-class output corresponding to the presence or absence of acromegaly.

**Figure 3:**
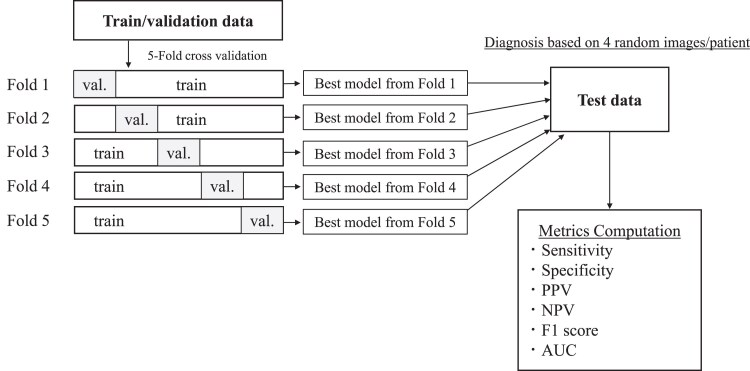
Training and evaluation scheme using 5-fold cross-validation. Schematic illustration of the training and evaluation procedures for AI. The training and validation data are divided into five folds for 5-fold cross validation. One subset was used for validation (val.) for each fold. The remaining four subsets were used for training. The best-performing model in each fold was selected based on the validation performance and was subsequently evaluated using independent test data. Diagnostic metrics, including sensitivity, specificity, positive predictive value (PPV), negative predictive value (NPV), F1 score, and area under the curve (AUC), were computed based on the test results.

In the model construction phase, the fully connected layer of ResNet-50 was removed, and a dropout layer (*P* = .2), followed by a fully connected layer for binary classification, was added (Fig. 2). The size of the input image was set to 448 × 448 pixels. As the image orientation was standardized across the dataset, we avoided vertical flips and large rotations during augmentation to preserve anatomical consistency, which is essential for the accurate identification and analysis of specific structures and to facilitate image-to-image comparisons for both human readers and automated systems. Data augmentation during training included random resized cropping (scale range: 0.95-1.1), random horizontal flipping, and normalization based on the mean and standard deviation of the ImageNet. The loss function was set to cross-entropy loss, and optimization was performed using stochastic gradient descent with a learning rate of 0.0001 and momentum of 0.9. The batch size was set to 16, the number of epochs was set to 40, and 5-fold cross-validation was conducted for training and validation. The best model for each fold was selected and saved based on the accuracy of the validation dataset (Fig. 3). Although Convolutional Neural Networks (CNNs) are powerful tools for image analysis, a single supervised CNN is prone to overfitting. To improve the performance and reliability, we performed 5-fold cross-validation and applied ensemble learning by aggregating predictions from models trained on different folds and initializations. This approach reduces variance and increases robustness to site- or artifact-specific patterns, thereby mitigating the key limitations of individual CNNs (26).

### Evaluation metrics

An ensemble prediction approach was used to assess the model performance by leveraging the availability of multiple hand images per individual. Four images were randomly selected for each patient ID, and the final probability score was obtained by averaging the softmax outputs of an ensemble of five ResNet-50 models. Classification was performed by applying an optimal threshold to the average probability. The optimal decision threshold was determined by maximizing the Youden index, which was calculated as follows: Youden index = Sensitivity + Specificity − 1. ROC curves were generated by plotting the true positive rate (sensitivity) against the false positive rate (1-specificity), and the threshold that yielded the highest Youden index was selected. The area under the ROC curve (ROC-AUC) was calculated to quantify the overall discriminatory ability of the models. Precision–recall curves were also constructed, and the area under the precision–recall curve (PR-AUC) was calculated to further evaluate model performance. The performance metrics included sensitivity, specificity, positive predictive value (PPV), negative predictive value (NPV), and F1-score, all of which were calculated from confusion matrices that compared the predicted labels with the ground truth. In addition, 95% confidence intervals (CI) for sensitivity, specificity, PPV, NPV, F1-score, ROC-AUC and PR-AUC were estimated using a bootstrap resampling approach. Calibration of the predicted probabilities was evaluated using a calibration plot. For the test dataset, predicted probabilities were grouped into 5 bins, and the mean predicted probability within each bin was plotted against the corresponding observed event rate to assess the agreement between predicted and observed risks. Furthermore, Gradient-weighted Class Activation Mapping (Grad-CAM) was applied to the selected test images to visualize the regions driving the model's decisions and confirm that attention was focused on the hand rather than the background or sleeves. All evaluations were performed using scikit-learn (https://scikit-learn.org/stable/), and ROC curves and Calibration Plot were plotted using Matplotlib (https://matplotlib.org/).

### Comparison with endocrinologists

An experiment was conducted by experienced board-certified endocrinologists to evaluate the efficacy of the proposed model. The endocrinologists assessed the hand images in the test dataset. The participating endocrinologists were affiliated with medical facilities distinct from those of the patients in the test dataset and were not involved in the dataset preparation or model development. Each endocrinologist independently conducted the reading experiments in separate environments. They were tasked with classifying 138 individuals from the test dataset into either the “acromegaly group” or the “control group.” Four images were randomly selected for evaluation for each patient.

### Subgroup analyses

We conducted subgroup analyses by further stratifying the test dataset into several groups. For each subgroup, we calculated the ROC-AUC, sensitivity, specificity, PPV, NPV, and F1-score.

**Age:** Patients in the test dataset were divided into two groups (younger and older) based on the median age, and model performance was evaluated in each group.

**Remission status:** Performance was assessed separately in patients with and without remission.

**Sex:** Performance was evaluated separately in male and female patients.

**Facility:** Performance was evaluated for each participating facility. Since one of the three facilities in the test dataset included only patients with acromegaly, we did not perform a subgroup analysis for that facility.

### Statistical analysis

The normality of the data was evaluated using the Shapiro–Wilk test. In the case of a normal distribution, the difference between the two groups was determined using Student's *t*-test, and the data were reported as the mean ± standard deviation. In the case of a non-normal distribution, the difference between the two groups was determined using the Mann–Whitney *U* test, and the data were reported as medians (interquartile range). For frequency comparisons, Fisher's exact test was used for 2 × 2 contingency tables; otherwise, the chi-square test was applied. Statistical significance was set at *P* < .05. Data were analyzed using GraphPad Prism software (version 10.4.2; GraphPad Software, Boston, MA, USA).

## Results

### Datasets

**Figure 4:**
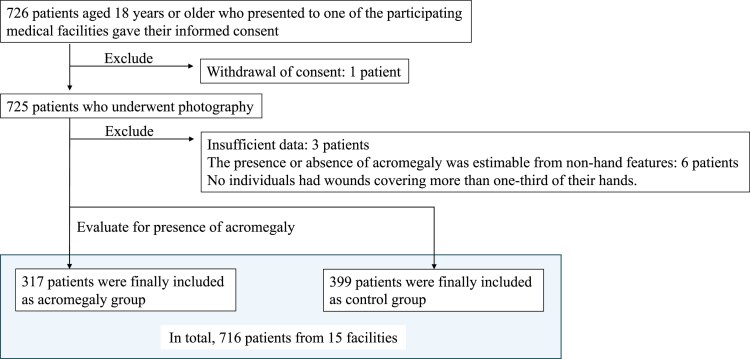
Flow diagram of participant enrollment and group allocation. Flowchart showing participant enrollment, exclusion, and final group allocation.

A total of 725 patients underwent manual image acquisition. Of these, 6 patients were excluded because non-hand elements were visible in the images, allowing the identification of the patient's group, thereby revealing the presence or absence of acromegaly. Three additional patients were excluded because of image data errors; these files could not be accessed or loaded properly at the central research facility. None of the participants met the exclusion criteria of wounds covering more than one-third of the hand. Finally, 716 patients (317 with acromegaly and 399 controls) were included in the dataset (Fig. 4).

**Figure 5:**
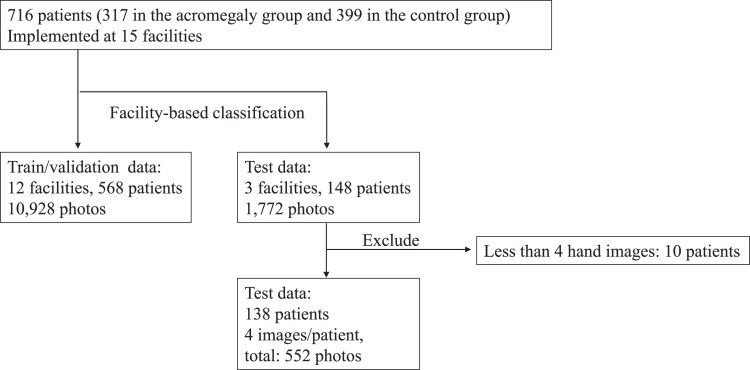
Dataset division according to the facility for model development and evaluation. Flowchart depicting the allocation of the 716 participants (317 in the acromegaly group and 399 in the control group) across 15 facilities. Facility-based classification was performed. Among the test data, 10 patients with fewer than four hand images were excluded, resulting in 138 patients in the final test data.

These 716 patients were divided into approximately 80% for the training/validation dataset and 20% for the test dataset based on the medical facilities to which they belonged. Moreover, 568 patients from 12 facilities were assigned to the training/validation dataset, and 148 patients from three facilities were assigned to the test dataset (Fig. 5).

### Clinical characteristics of the patients

The demographic and clinical characteristics of the patients were summarized in Table 1. No significant differences were observed in age or sex distribution between the acromegaly and control groups, with median ages of 57 and 56 years in the acromegaly and control groups, respectively. Of the 310 patients with acromegaly and available treatment history, 254 (81.9%) had previously undergone surgery. At the time of image, 184 patients (59.3%) were considered to have achieved biochemical remission based on clinical and laboratory criteria. The mean GH concentration at the time of imaging was 7.17 ± 27.68 ng/mL, and the IGF-I SDS was 1.30 ± 2.88. These values were lower than those at the time of diagnosis, where the mean GH levels were 40.11 ± 100.82 ng/mL and IGF-I SDS was 7.24 ± 3.04. This substantial reduction reflects the effects of treatment and disease control over time in a significant proportion of patients with acromegaly. In addition, 191 participants (47.9%) were classified as controls based solely on expert clinical adjudication without measurement of IGF-I.

**Table 1:** Baseline clinical characteristics of patients with acromegaly and control participants

	Acromegaly	Control	*P*-value
	(n = 317)	(n = 389)	
Sex, male/female	148/169	181/208	.967
Age [IQR] (years)	57.0 [46-69]	56.0 [44-69]	.359
BMI (kg/m^2^)	24.68 ± 4.00	—	—
History of surgery for acromegaly, yes/no	254/56	—	—
Medical treatment for acromegaly, yes/no	150/57	—	—
Postoperative remission status, remission/non-remission/uncertain	184/109/3	—	—
Duration since remission, ≤1 year/ > 1-5 years/ > 5-10 years/ > 10 years	11/71/52/51	—	—
Random GH levels at diagnosis (ng/mL)	40.11 ± 100.82	—	—
IGF-I levels at diagnosis (ng/mL)	701.17 ± 338.47	—	—
IGF-I SDS at diagnosis	7.24 ± 3.04	—	—
Random GH levels at photo (ng/mL)	7.17 ± 27.68	—	—
IGF-I levels at photo (ng/mL)	227.46 ± 220.42	—	—
IGF-I SDS at imaging	1.30 ± 2.88	—	—
Hydrocortisone replacement therapy, yes/no	29/281	—	—
Gonadotropin replacement therapy, yes/no	8/302	—	—
Levothyroxine replacement therapy, yes/no	28/282	—	—

Data are presented as mean ± standard deviation (SD) for continuous variables or as counts for categorical variables.

Abbreviations: BMI, body mass index; GH, growth hormone; IGF-I, insulin-like growth factor-I; IQR, interquartile range; SDS, standard deviation score.

*P*-values were calculated for sex and age comparisons between groups. Clinical data and specific measurements were partially missing in some cases, except for sex and age, which were available for all participants.

The patients were divided into 2 groups based on the participating institutions: training/validation and test datasets (Fig. 5). No significant differences were observed in age, sex distribution, or proportion of patients with acromegaly and control participants between the 2 datasets (Table 2).

**Table 2:** Baseline characteristics of participants in the train/validation and test data

	Train/validation	Test	*P*-value
	(n = 568)	(n = 138)	
Group, acromegaly/control	260/308	57/81	.344
Sex, male/female	272/296	57/81	.164
Age [IQR] (years)	56.5 [44-69]	57.0 [46-70]	.386
BMI (kg/m^2^)	24.2 [22.1-26.7]	23.5 [21.5-26.9]	.782
History of surgery for acromegaly, yes/no	202/51	52/5	.**043**
Medical treatment for acromegaly, yes/no	124/126	26/31	.587
Postoperative remission status, remission/non-remission	153/53	31/15	.342
Duration since remission, ≤1 year/ > 1-5 years/ > 5-10 years/ > 10 years	10/56/44/44	1/5/8/7	.652
Random GH levels at diagnosis (ng/mL)	14.90 [7.20-33.30]	22.49 [11.73-45.13]	.080
IGF-I levels at diagnosis (ng/mL)	614.0 [463.0-849.0]	705.0 [445.3-972.3]	.392
IGF-I SDS at diagnosis	6.90 [5.20-8.82]	6.10 [4.70-8.40]	.164
Random GH levels at photo (ng/mL)	1.66 [0.70-4.59]	1.15 [0.72-2.80]	.118
IGF-I levels at photo (ng/mL)	154.0 [118.0-238.0]	142.0 [103.0-236.0]	.198
IGF-I SDS at imaging	0.57 [−0.30 to 2.20]	0.20 [−0.70 to 1.89]	.120
Hydrocortisone replacement therapy, yes/no	22/231	7/50	.401
Gonadotropin replacement therapy, yes/no	7/246	1/56	.663
Levothyroxine replacement therapy, yes/no	21/232	7/50	.344

Data are presented as mean ± standard deviation (SD) for continuous variables or as counts for categorical variables.

Abbreviations: BMI, body mass index; GH, growth hormone; IGF-I, insulin-like growth factor-I; IQR, interquartile range; SDS, standard deviation score.

*P*-values were calculated for sex and age comparisons between groups. All columns other than Group (disease acromegaly/control), age, and sex represent variables measured only in participants with acromegaly. For these variables, clinical data and specific measurements were partially missing. *P*-values < .05 are shown in bold.

### Performance metrics of the AI model and endocrinologists

**Figure 6:**
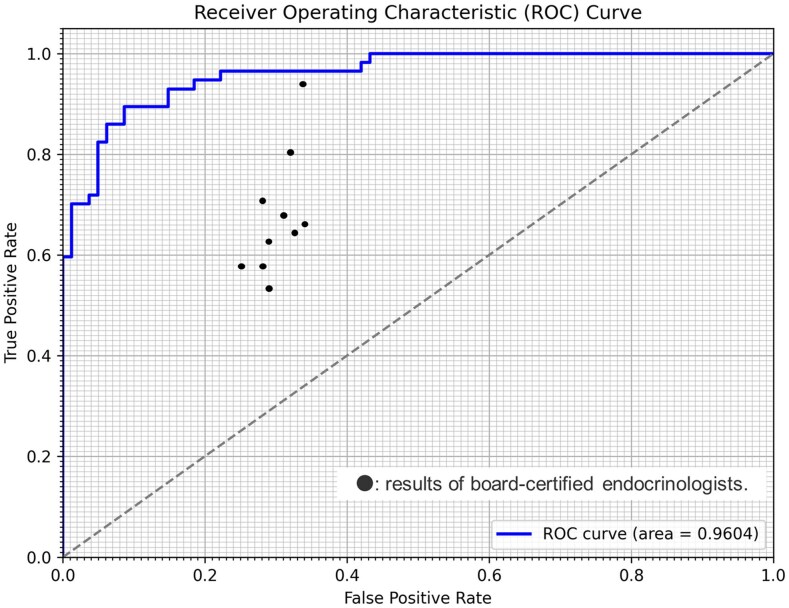
Receiver operating characteristic (ROC) curve of the AI model compared with board-certified endocrinologists. The ROC curve of the AI model for detecting acromegaly was plotted based on test data. The area under the curve (ROC-AUC) was 0.9604, indicating an excellent diagnostic performance. The black dots represent the sensitivity and false-positive rate (1-specificity) for each endocrinologist's prediction, corresponding to the performance metrics summarized in Table 3. The diagonal dashed line indicates the line of non-discrimination (AUC = 0.5).

**Figure 7:**
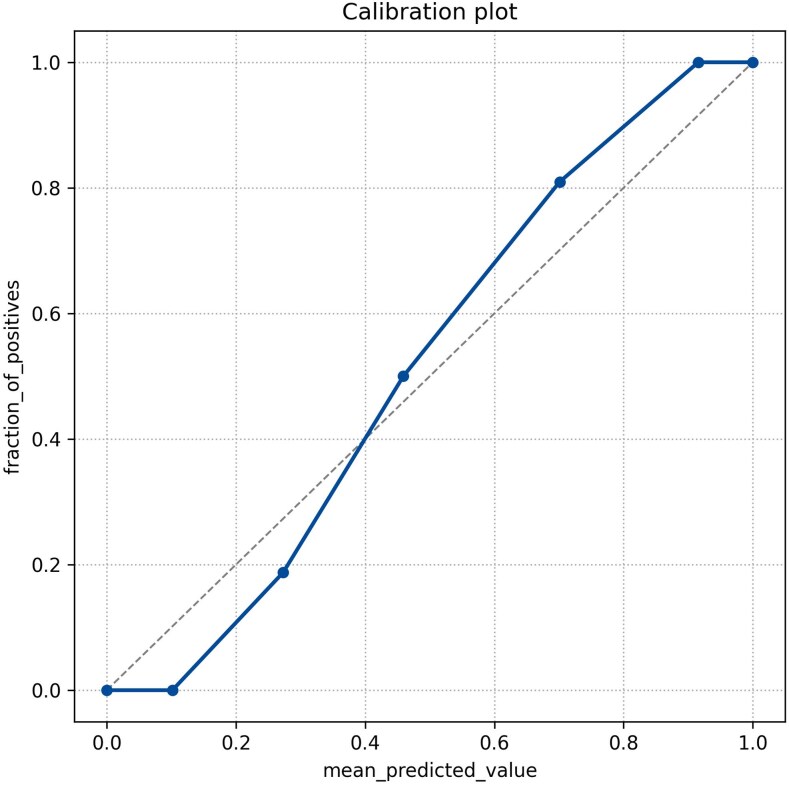
Calibration of the AI model on the test dataset. Calibration plot showing the relationship between the predicted probabilities and the observed fraction of positive cases. The solid blue line represents the model's predictions grouped into probability bins, and the grey dashed line indicates perfect calibration (predicted risk = observed risk).

Using this dataset, we developed a ResNet-50-based model. At the optimal decision threshold of 0.417, determined by maximizing the Youden index (0.81), the model achieved a sensitivity of 0.89 (95% CI 0.81-0.96) and specificity of 0.91 (95% CI 0.85-0.97). The model yielded a PPV of 0.88 (95% CI 0.79-0.95), NPV of 0.93 (95% CI 0.86-0.97), and F1-score of 0.89 (95% CI 0.82-0.94), indicating a well-balanced and reliable performance. The ROC-AUC was 0.96 (95% CI 0.93-0.98), indicating a potentially useful performance. The findings from the comparative analysis with endocrinologists conducted through a diagnostic image interpretation experiment are illustrated (Fig. 6 and Table 3). Compared with 10 endocrinologists with 9.8 (5-17) years of experience, the model demonstrated higher sensitivity and specificity. In addition, the PR-AUC was 0.95 (95% CI 0.91-0.98). The calibration plot (Fig. 7) showed that the predicted probabilities were generally consistent with the observed event rates across probability bins, with modest deviations at the lower and higher probability ranges, indicating reasonably good calibration of the model's probability estimates.

**Table 3:** Performance metrics of the AI model and endocrinologists for acromegaly detection

	AI model	E. 1	E. 2	E. 3	E. 4	E. 5	E. 6	E. 7	E. 8	E. 9	E. 10
Sensitivity (95% CI)	0.89 (0.81-0.96)	0.58	0.71	0.58	0.54	0.63	0.68	0.81	0.65	0.67	0.94
Specificity (95% CI)	0.91 (0.85-0.97)	0.75	0.72	0.72	0.71	0.71	0.69	0.68	0.67	0.66	0.66
PPV (95% CI)	0.88 (0.79-0.95)	0.68	0.53	0.61	0.65	0.54	0.46	0.38	0.42	0.35	0.28
NPV (95% CI)	0.93 (0.86-0.97)	0.65	0.85	0.69	0.60	0.78	0.85	0.94	0.84	0.88	0.99
F1 score (95% CI)	0.89 (0.82-0.94)	0.63	0.61	0.60	0.59	0.58	0.55	0.51	0.51	0.46	0.43

Comparison of diagnostic performance between the AI model and individual board-certified endocrinologists (endocrinologist 1-10). All values are shown as raw scores without percentage conversion. The area under the receiver operating characteristic curve (ROC-AUC) for the AI model was 0.96 (95% CI 0.93-0.98).

Abbreviations: AI, artificial intelligence; E., endocrinologist; AUC, area under the receiver operating characteristic curve; CI, Confidence Interval.

**Figure 8:**
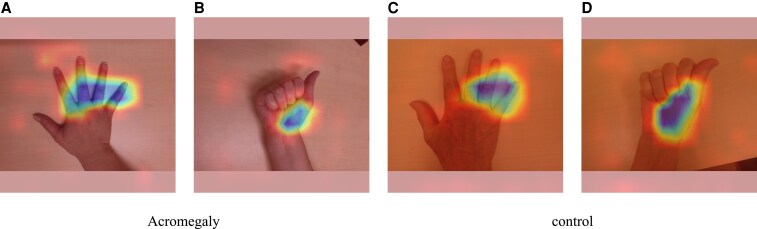
Representative hand images from test data. Representative examples of test data and Grad-CAM (A–D: acromegaly dorsal hand, acromegaly clenched fist, control dorsal hand, and control clenched fist, respectively), outputting a heatmap of the parts of the image that had the greatest impact on the prediction.

A heatmap generated using Grad-CAM was overlaid on a representative test image to highlight the areas that most influenced the predictions of the AI model (Fig. 8). These visualizations suggest that the model makes decisions based on the hand morphology recognition rather than the background or other non-hand cues.

### Subgroup analyses

Subgroup analyzes are summarized in Table 4. Across age strata, diagnostic performance was broadly comparable, with ROC-AUCs of 0.96 and 0.95, sensitivities of 0.89 and 0.86, and specificities of 0.90 and 0.95 in the younger (<57 years) and older (≥57 years) groups, respectively., performance remained stable when stratified by remission status, with ROC-AUCs of 0.94 in the remission group and 0.97 in the non-remission group, and F1-scores of 0.81 and 0.84, respectively. Stratification by sex also showed consistently high accuracy, with ROC-AUCs of 0.97 and 0.94 and F1-scores of 0.93 and 0.84 in male and female patients, respectively. Facility-specific analyses of the two test sites showed comparable ROC-AUCs (0.90 for both facilities A and B), although PPV and NPV varied between facilities (PPV 0.44 and NPV 0.98 for facility A; PPV 0.93 and NPV 0.73 for facility B), likely reflecting differences in disease prevalence and case mix.

**Table 4:** Performance metrics of the AI model: subgroup analyses

	Age		Remission status		Sex		Facility (test data)	
	<57y	≥57y	Remission	Non-remission	Male	Female	A	B
ROC-AUC	0.96	0.95	0.94	0.97	0.97	0.94	0.90	0.90
Sensitivity	0.89	0.86	0.90	0.91	0.93	0.90	0.89	0.90
Specificity	0.90	0.95	0.88	0.93	0.93	0.86	0.86	0.80
PPV	0.86	0.93	0.74	0.78	0.93	0.79	0.44	0.93
NPV	0.93	0.91	0.96	0.97	0.93	0.94	0.98	0.73
F1 score	0.88	0.89	0.81	0.84	0.93	0.84	0.59	0.92

All values are shown as raw scores without percentage conversion. Since one of the 3 facilities in the test dataset included only acromegaly group, we did not perform a subgroup analysis.

Abbreviations: AI, artificial intelligence; ROC-AUC, area under the receiver operating characteristic curve.

### Error analysis

There were seven false-positive cases in which the AI model judged the images as positive, although the individuals belonged to the control group. These patients had a median age of 48 years and were relatively young; 5 (71.4%) were male. In 6 of these 7 cases (85.7%), the correct classification rate among endocrinologists was ≥80%.

Conversely, there were 6 false-negative cases in which the AI model judged the images as negative, although the individuals had acromegaly. Their median age was 59 years, all were female, and all had a history of pituitary surgery. The two non-remission cases were receiving medical therapy. In 5 of the 6 false-negative cases (83.3%), the correct classification rate among endocrinologists was ≤20%, indicating that these cases were also challenging for endocrinologists.

## Discussion

This study represents the first attempt to develop a detection tool for acromegaly that preserves patient privacy by avoiding exposure to identifiable features, such as facial characteristics or fingerprints. Although the characteristic visual findings of acromegaly, such as hand abnormalities and the so-called fist sign, have been recognized and utilized based on expert opinions in clinical practice, these features have not been formally defined or systematically evaluated. In this study, we proposed standardized definitions for the “dorsal hand sign” and the “fist sign,” and showed that AI-based diagnostic support using photos of these signs.

The AI model developed in this study demonstrated strong diagnostic performance using anonymized images. Trained on a large dataset of over 11 000 images, the model achieved metrics that, in some cases, particularly in terms of specificity, exceeded those of endocrinologists experienced in managing acromegaly. A Grad-CAM heatmap shows the attention of the model, suggesting that it may base its predictions on the finger joints, fingernails of a clenched fist, and the region near the base of the thumb. Compared to prior approaches, such as a study using hand images that include identifiable features such as fingerprints, our model adopts a privacy-by-design strategy. It analyzes the dorsal hand sign and fist sign and achieves diagnostic performance comparable to prior studies. Consequently, by reducing compliance burdens and legal risks, it sidesteps many of the regulatory and ethical hurdle. This, in turn, fosters greater acceptance among patients and providers and makes deployment feasible in non-specialist settings (eg, primary care and telehealth) and public health screening tools (medical checkups and health apps). This practical advantage enables earlier detection at the initial points of contact, offers a tangible solution to reduce diagnostic delays, and translates AI research into a public health tool.

Although the AI model has demonstrated promising results in detecting acromegaly using hand images, its limitations must be acknowledged. Endocrinologists do not rely solely on visual cues to diagnose acromegaly. They considered various factors, including changes in voice, facial expressions, and other physical and biochemical markers, as well as medical history and comorbid conditions. They evaluated the temporal changes in the physical findings. This comprehensive assessment approach enables a nuanced and accurate diagnosis. Although valuable as a screening tool, the AI model may not capture the full spectrum of clinical manifestations and should be viewed as a complement to expert clinical judgment rather than a replacement.

Although a previous study using 992 hand images demonstrated high diagnostic performance with a sensitivity of 0.983, specificity of 0.920, and F1-score of 0.974 (27), its dataset differed from ours; more than half of the images were from patients with acromegaly, and palmar-view hand images were included. This study supports our hypothesis that acromegaly can be diagnosed using hand images alone with an accuracy comparable to that reported for facial image-based AI diagnosis (F1-score 0.92, sensitivity 0.93, and specificity 0.93) (16). Our findings suggest that deep learning applied to anonymized images can maintain high diagnostic performance.

In clinical practice, particularly for rare diseases with characteristic external manifestations, early diagnosis often relies on visual assessment by endocrinologists alone. Previous studies have indicated that visual inspection alone has limitations in accurately diagnosing these conditions (28). Moreover, general practitioners and nonspecialists may struggle to recognize subtle external signs, particularly during brief consultations. Although executive health programs and routine medical checkups play important roles in the early detection of diseases, identifying such conditions based solely on subtle external signs during these evaluations remains challenging (10, 29). In practice, only a limited number of patients with this condition have been identified using these methods. The widespread use of face masks during the Coronavirus Disease 2019 (COVID-19) pandemic has further complicated visual assessments by obscuring critical facial features (30). Despite these limitations, this diagnostic model may facilitate early detection and timely referral to endocrinologists.

This study highlights the potential of AI to enable accurate disease identification based solely on external physical traits, without compromising patient anonymity. The ability of such a model to assist healthcare providers who may not have specialized training represents a significant step forward in diagnostic accuracy and in promoting equitable healthcare delivery. By facilitating disease detection while respecting privacy, this tool may bridge disparities in expertise and healthcare access, supporting the broader and earlier identification of affected individuals. Furthermore, this study is the first to demonstrate that the dorsal hand sign and fist sign (21) may serve as clinically useful physical markers for this condition. Although this model leveraged both signs in its training, separate analyses to evaluate the relative diagnostic contributions of each sign were not performed. Such comparisons may represent a valuable direction for future research.

AI-based facial recognition technology is rapidly advancing. For example, DeepGestalt is an AI-driven facial diagnostic system that is currently under development (31). It analyzes facial images to prioritize candidate genetic syndromes. However, these design features raise questions regarding their reproducibility and generalizability beyond facial phenotypes. In contrast, our study used hand images and framed the task as the binary detection of a specific disease, thereby minimizing the reliance on personally identifiable information. Given the limitations of this model in single-disease binary classification, the next step is to extend it to additional conditions using insights from this study. In this context, while the immediate focus of this study was to detect acromegaly, the underlying principles of this approach could be extended to diseases identifiable through dorsal hand signs, fist signs, or nail signs, such as rheumatoid arthritis (32, 33), anemia, and finger clubbing, thereby enhancing its practical utility in clinical practice. AI-based models can complement clinical expertise, reduce diagnostic oversight, and enable earlier intervention, thereby contributing to the prevention of complications (34). These advantages may be further enhanced by the ability of such models to function effectively without an overreliance on personally identifiable information (35).

Although the dorsum of the hand and clenched fist are relatively safe, we adopted a privacy-by-design approach by focusing exclusively on these views and excluding palmar surfaces and facial features. Residual re-identification risks from anatomical patterns such as venous structures and knuckle creases cannot be completely eliminated but are likely to be low in this dataset because of standardized image acquisition, uniform backgrounds, and the absence of obvious personal identifiers. The physical signs of acromegaly are most evident on the face, hands, and feet. Although not among these canonical sites, the forearm and lower leg, areas associated with a lower re-identification risk, may warrant limited exploration, as relevant features are likely uncommon. Such privacy-enhanced expansion would probably require a larger dataset than the current model, along with flexible adjustments to the imaging conditions and data augmentation.

This study had certain limitations that should be acknowledged. First, more than half of the patients with acromegaly were evaluated after achieving remission, and their characteristic physical features may have diminished over time, potentially affecting the performance of the model. Although cartilaginous and articular changes caused by acromegaly may be partially reversible early on, they often persist for a long time (36, 37). Thus, valid results can still be obtained even when the dataset includes many patients with remission. Second, although the control participants were considered unlikely to have acromegaly based on the judgment of endocrinologists or pituitary neurosurgeons, the exclusion of the disease was not systematically confirmed in all participants according to the established diagnostic criteria. Experts judged some participants in the control group to have no acromegaly, without GH/IGF-I measurements. Although the likelihood was considered low, we cannot completely rule out the possibility that undiagnosed patients with acromegaly were included, which may have influenced the specificity of the results. In such cases, individuals with unrecognized acromegaly who were classified as controls and tested positive would have been counted as false positives, potentially biasing the estimated specificity toward an underestimation of the true value. Nonetheless, the credibility of control selection remains substantial, given that endocrinologists or pituitary neurosurgeons screened individuals who were deemed highly unlikely to have acromegaly based on clinical judgment. Third, the proportion of patients with acromegaly in this dataset was higher than that observed in real-world clinical settings, which may have resulted in differences between the observed and actual diagnostic performance of the model in routine practice. Fourth, there may be a selection bias, as the patients included in this study may not represent the full spectrum of patients with acromegaly encountered in clinical practice. Finally, in terms of real-world implementation, the low prevalence of acromegaly, testing costs, downstream workups from false positives (with attendant psychosocial burdens), and constrained healthcare resources may limit the utility of indiscriminate screening. A comprehensive analysis incorporating economic and social factors is essential before recommending such programs. Moreover, because the model was trained exclusively on Japanese patients in specialist centers, its generalizability to other ethnic groups and to community or primary-care settings remains uncertain, and external validation in multiethnic, non-specialist cohorts will be essential to confirm its performance and applicability.

This study had several strengths. This demonstrates the potential of using privacy-conscious deep learning on hand images for the automatic detection of acromegaly, achieving a high diagnostic accuracy that outperforms endocrinologists. The ability of the model to detect subtle physical signs of acromegaly from anonymized hand images without relying on identifiable facial features represents a significant advancement in the development of ethical and practical AI-based screening tools for diseases with distinct physical signs. As a nationwide collaborative study involving 15 medical facilities across Japan, the dataset was divided on an institutional basis to rigorously evaluate its generalizability. As data collection and image acquisition were performed across multiple institutions with heterogeneous cameras, lighting conditions, and staff, the study reflects real-world variability, thereby strengthening the robustness of the model and supporting its clinical applicability.

In conclusion, this study demonstrated the feasibility of a privacy-preserving AI model for detecting acromegaly using hand images, achieving a diagnostic performance that surpasses that of experienced endocrinologists. This model may facilitate the detection and referral of patients in non-specialist settings. Further validation in diverse populations and applications for other visually detectable conditions is warranted.

## Data Availability

Some or all datasets generated and/or analyzed during this study are not publicly available can be obtained from the corresponding author upon reasonable request.
